# Bibliographical Notices

**Published:** 1844-05-18

**Authors:** 


### BIBLIOGRAPHICAL NOTICES.

*Outlines of Pathology and Practice of Medicine.* By WILLIAM PULTENEY ALISON, M. D., F. R. S. E., Fellow and late President of the Royal College of Physicians of Edinburgh, &c. &c. Philadelphia: Lea & Blanchard, 1844. 1 vol. 8vo. pp. 424.

This is a work of no ordinary character;—one of those rare productions that exhibit on every page evidences of a well stored and thoroughly disciplined mind. Its distinguished author is the Professor of the Practice of Medicine in the University of Edinburgh, and this volume seems to have been prepared to be used as a text book by those attending his lectures; but however well it may be adapted for that object, it may be read with equal advantage by the oldest and best informed of the profession. We have seldom met with a medical work so remarkable for condensation. Although a great amount of matter is comprised in a comparatively small space, still nothing is incomplete, nothing obscure; and the language is as smooth as if elegance of style, and not mere brevity of expression, was the object to be attained. The author, from a consideration of the facts best ascertained in regard to the nature, progress, and symptoms of diseases, and the effects of remedies upon them, has endeavoured to simplify as far as possible, both the diagnostic marks of diseases, and the practical rules for their treatment—dwelling only upon those, an accurate knowledge of which may be acquired without much difficulty, and on which it has appeared to him, in practice, we can rely with most confidence. We are glad to find that he concurs with his illustrious countryman, Cullen, in maintaining that, "at all times the Practice of Medicine has been, and still is with every person (of intelligence) founded more or less upon certain principles established by reasoning." To abandon this ground, is to embrace empiricism in all its protean forms. In the principles inculcated by our author, we find nothing that is not accordant with the best established facts in pathology and therapeutics; whilst the phenomena exhibited by disease, and the effects of remedies, are often presented in an aspect so new and yet so consistent and natural, as to forcibly impress the mind with relations not generally observed. We regret that we cannot offer to our readers an analysis of the work—it is, in fact, itself an analysis of the subjects on which it is written, which could hardly be more condensed or better explained.

---

*The Practice of Medicine. A Treatise on Special Pathology and Therapeutics.* By ROBLEY DUNGLISON, M. D., Professor of the Institutes of Medicine, etc., in Jefferson Medical College, Philadelphia; Lecturer on Clinical Medicine, and Attending Physician at the Philadelphia Hospital; Secretary of the American Philosophical Society, etc. etc. *Second Edition.* In 2 volumes. Octavo. Philadelphia: Lea & Blanchard, 1844.

Two years have elapsed since the appearance of the first edition of this work. In that short period, one large edition has been sold and another printed! This is substantial evidence of the opinion of the profession in this country in regard to its merits; nor has there been any lack of praise from the journalists of Europe. Those who have read the successive editions of the Author's works know that they are not mere reprints, one of another; but that the last is always better than the preceding. No author is at greater pains, by additions and careful revision, to render his productions faithful exponents of the latest improvements on the subjects on which he writes. The present work is emphatically of this character. Some additional articles have been introduced, some errors incident to a first edition corrected, and on every topic the latest facts and most approved views of pathology and therapeutics are introduced and discussed.

---

*Pathological Hæmatology. An Essay on the blood in disease.* By G. ANDRAL, *Professor of General Pathology and Therapeutics in the University of Paris, etc. Translated from the French by* J. F. MEIGS, M. D., and ALFRED STILLE, M. D. Philadelphia, Lea and Blanchard, 1844, 8vo. pp. 129.

This is an exceedingly interesting little work, the size of which, in fact, bears no proportion to the labour which it must have caused the author to produce it. Solidism, Humorism, and Vitalism, for a long time divided the cultivators of medical science, each to the exclusion of the others, without its occurring to the several advocates that all in turn might be right. Now, however, the truly philosophical investigators of pathology, seek for the manifestations of disease equally in the fluids and the solids, and in their dynamical condition. M. Andral has been engaged for a long time, alone, and co-operating with Messrs. De La Fond and Gavarret, in investigating the properties of the blood in disease. The present Essay may be considered as the conclusions at which he has arrived in the course of these investigations. It is divided into two chapters. Chapter I., "treats *of the best method to pursue in the study of pathological hæmatology.*" Under this head he notices briefly the distinguished physicians who in the earlier periods of our profession directed their attention to the blood in disease, points out the erroneous manner in which the subject was regarded, speaks of its condition in a physiological state, in the triple aspect of its physical properties, its chemical composition, and its microscopic constitution. Having established a standard condition in these respects, which, however, may vary within certain limits from other causes than disease, he proceeds in the succeeding chapter to indicate, so far as observation has gone, the changes in these respects which take place in certain morbid conditions. Chapter II., after some general remarks "*on the blood in diseases*," is divided into Eight articles:

I. Of the blood in Plethora,
II. Of the blood in Anæmia,
III. Of the blood in Pyrexia,
IV. Of the blood in Phlegmasiæ,
V. Of the blood in Hemorrhages,
VI. Of the blood in Dropsies,
VII. Of the blood in certain diseases commonly called organic,
VIII. Of the blood in the Neuroses,

We have thus enumerated the subjects embraced in this Essay, that our readers may judge of the extent of ground which it covers. It contains indeed, a great amount of matter, well deserving of the attention of all who would fully comprehend the modifications that occur in the blood, as the cause or effect of disease.

---

*The Cyclopædia of Practical Medicine, Part III.*

The Editor and Publishers seem determined to bring this work to a close as speedily as possible. We have now three *parts* in about as many weeks. The present number (part iii.) contains two articles, from Bathing to Cholera, and is fully equal to those which have preceded it.

